# Research progress of the chemokine/chemokine receptor axes in the oncobiology of multiple myeloma (MM)

**DOI:** 10.1186/s12964-024-01544-7

**Published:** 2024-03-12

**Authors:** Jun Du, Zheng Lin, Xue-Hang Fu, Xiao-Ran Gu, Guang Lu, Jian Hou

**Affiliations:** 1 Department of Hematology, Renji Hospital, School of Medicine, Shanghai Jiao Tong University, Shanghai, 200127 China; 2 Shanghai Jiao Tong University School of Medicine, Shanghai, 200025 China; 3 Department of Hematology, Shengli Oilfield Central Hospital, Dongying, 257099 China

**Keywords:** Chemokines, Multiple Myeloma (MM), CAR-T cell therapy

## Abstract

**Background:**

The incidence of multiple myeloma (MM), a type of blood cancer affecting monoclonal plasma cells, is rising. Although new drugs and therapies have improved patient outcomes, MM remains incurable. Recent studies have highlighted the crucial role of the chemokine network in MM’s pathological mechanism. Gaining a better understanding of this network and creating an overview of chemokines in MM could aid in identifying potential biomarkers and developing new therapeutic strategies and targets.

**Purpose:**

To summarize the complicated role of chemokines in MM, discuss their potential as biomarkers, and introduce several treatments based on chemokines.

**Methods:**

Pubmed, Web of Science, ICTRP, and Clinical Trials were searched for articles and research related to chemokines. Publications published within the last 5 years are selected.

**Results:**

Malignant cells can utilize chemokines, including CCL2, CCL3, CCL5, CXCL7, CXCL8, CXCL12, and CXCL13 to evade apoptosis triggered by immune cells or medication, escape from bone marrow and escalate bone lesions. Other chemokines, including CXCL4, CCL19, and CXCL10, may aid in recruiting immune cells, increasing their cytotoxicity against cancer cells, and inducing apoptosis of malignant cells.

**Conclusion:**

Utilizing anti-tumor chemokines or blocking pro-tumor chemokines may provide new therapeutic strategies for managing MM. Inspired by developed CXCR4 antagonists, including plerixafor, ulocuplumab, and motixafortide, more small molecular antagonists or antibodies for pro-tumor chemokine ligands and their receptors can be developed and used in clinical practice. Along with inhibiting pro-tumor chemokines, studies suggest combining chemokines with chimeric antigen receptor (CAR)-T therapy is promising and efficient.

## Introduction

Multiple myeloma (MM) is a blood cancer of monoclonal plasma cells (PCs) characterized by hypercalcemia, renal insufficiency, anemia, or osteolytic lesions [1]. According to the epidemiological landscape of MM in 2022, though the global burden of MM varied from the country, the overall incidence of MM was increasing [2]. Thanks to new drugs such as immunomodulatory agents, proteasome inhibitors, monoclonal antibodies, and so on, the survival of MM patients has been significantly improved [3, 4]. However, MM is still an incurable disease. To develop effective treatments and manage MM patients, researchers should foster an overview of the pathological mechanism of MM cells. In addition to genic mutations in malignant cells, interactions between malignant cells and normal cells also contribute to the progression of MM.

Chemokines are a member of the cytokine superfamily with chemoattractant properties. According to the arrangement of amino-terminal cysteine (C) residues, chemokines are divided into four subfamilies: CXC, CC, XC, and CX3C subfamily. In the tumor microenvironment (TME), chemokines are secreted by different kinds of cells, including immune cells, tumor cells, and tumor-associated cells. Chemokines engage in immune cells’ activation, differentiation, proliferation, migration, and apoptosis and form a complex network in the immune system [5]. Malignant cells can evade apoptosis via secreting chemokines to recruit immunosuppressive cells, while immune cells also migrate to TME to attack tumors via chemokines.

Researchers have developed inhibitors, antibodies, or antagonists to block interactions between chemokine ligands and receptors as adjuvant therapy. CXCR4 antagonists such as plerixafor [6] and ulocuplumab [7] are safe and effective in combination with bortezomib. Plerixafor [8] and motixafortide [9] can improve the mobilization of stem cells. The success of CXCR4 antagonists indicates that targeting chemokines and their receptors is a potential strategy for managing MM. Therefore, insights into the network of chemokine ligands and receptors can contribute to the oncobiology of MM, which further improves treatment strategy and prognosis.

Thus, this review aims to summarize research on the chemokine network in MM in the past 5 years and discuss chemokines in the pathological progression of MM and potential therapy targeting related chemokines.

## Main text

### Chemokines as biomarkers

**Fig. 1:**
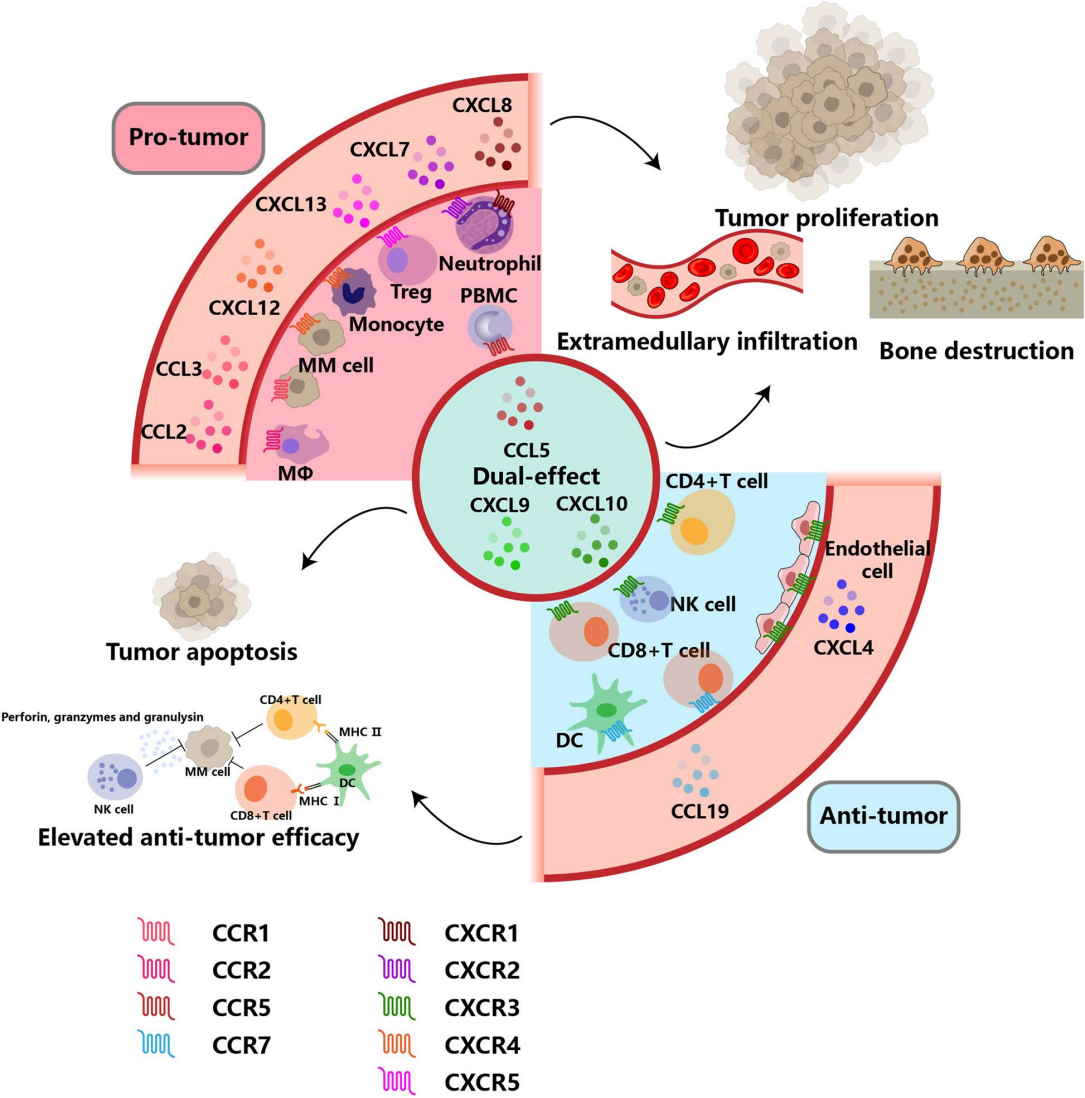
The chemokine network in multiple myeloma

**Fig. 2:**
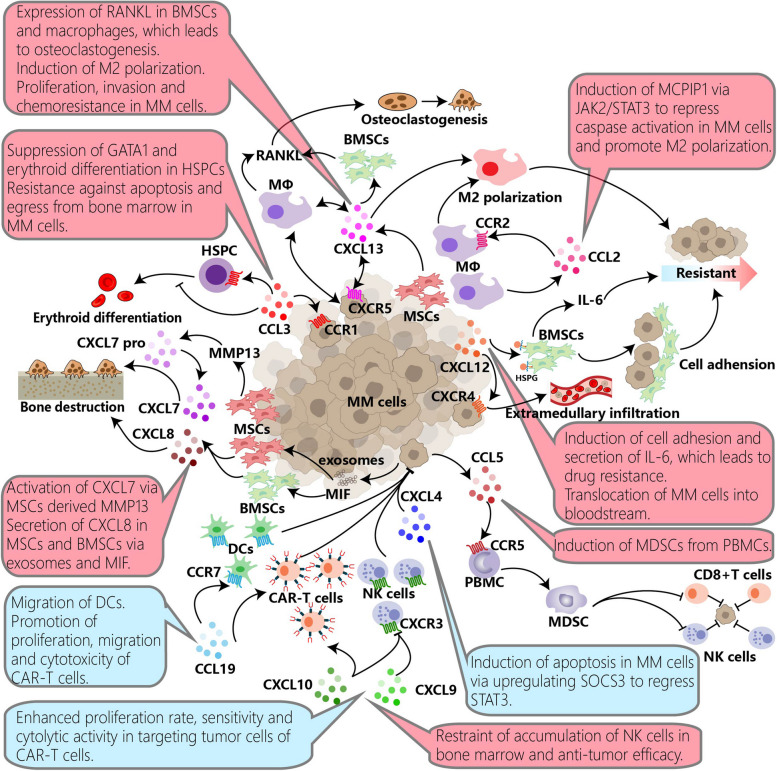
The function and mechanism of chemokines in multiple myeloma

According to previous studies, chemokines can serve as biomarkers and targets for various tumors, including hepatocellular carcinoma [10], endometrial cancer [11], and colorectal cancer [12]. Recent studies highlighted the role of chemokines in the pathological progression of MM (Fig. 1). On the one hand, chemokines participate in the body’s metabolism. Chemokines directly secreted or indirectly induced by malignant cells may disturb the normal function of chemokines and lead to several complications in MM. The breakup of chemokine balances can cause common complications, including bone destruction and anemia, in MM. It has been suggested that a higher level of activated osteoclastic chemokines, such as CXCL7, aggravated bone destruction in MM [13]. Besides, MM cells can secret CCL3 to disrupt erythrocyte differentiation and cause anemia [14]. These complications caused by the abnormal level of chemokines exacerbate the disease and the pain of patients. On the other hand, chemokines take part in the formation of the immune environment, which impacts the clearance of malignant cells. Malignant cells can utilize chemokines to recruit immune cells to protect themselves from apoptosis [15]. The level of chemokines in MM patients also influences the proliferation, migration, and recruitment of immune cells, which is associated with the effectiveness of chimeric antigen receptor (CAR)-T therapy [16, 17]. MM cells can also utilize chemokines to migrate, which causes extramedullary infiltration and exacerbates patients’ burden [18]. Experts proposed chemokines could be potential biomarkers for MM to predict progression and prognosis. Here, we will delve into chemokines’ function and significance in MM (Fig. 2).

Chemokines are mainly comprised of the CXC subfamily and CC subfamily chemokines. According to their function in MM, they can be divided into pro-tumor, anti-tumor, and dual-effect chemokines. Pro-tumor chemokines can promote extramedullary infiltration, bone destruction, and tumor proliferation by interaction with MΦs, monocytes, neutrophils, Tregs, or PBMCs. Contrastingly, anti-tumor chemokines can bind to their receptors to induce NK cells, CD8 + T cells, DCs, or endothelial cells to enhance their anti-tumor efficacy and tumor apoptosis. Or some chemokines, including CCL5 and CXCL10, are double-edge swords in MM. MΦs, macrophages; Tregs, regulatory T cells; PBMCs, peripheral blood mononuclear cells; NK cells, natural killer cells; DCs, dendritic cells; MM, multiple myeloma.

#### CC chemokine/receptor subfamily

CC chemokines are a group of cytokines with an N-terminal CC domain. These chemokines reveal both anti-tumor and pro-tumor functions in MM. While some may lead to deteriorated anemia, chemoresistance, and tumor dissemination, others may aid in the recruitment of dendritic cells (DCs) essential for the immune system to attack malignant cells.

To resist apoptosis induced by chemical drugs, MM cells may attempt to recruit tumor-associated macrophages (TAMs) or myeloid-derived suppressor cells (MDSCs) to form TME. Newly published studies suggest that chemokines CCL2, CCL3, and CCL5, along with their receptors, participate in the formation of chemoresistance in MM.

CCL2, known as monocyte chemoattractant protein-1 (MCP-1), is mainly secreted by monocytes, macrophages, and DCs [19]. It interacts with CCR2 to promote tumor growth and progression through various mechanisms. Previous studies have shown that the CCL2-CCR2 axis is related to increased angiogenesis, recruitment of immunosuppressive cells, and the proliferation and survival of malignant cells [20]. Recent research suggests that CCL2 is linked to M2 polarization and contributes to MM chemoresistance [15]. MM cells upregulate CCL2 expression in macrophages, further upregulating MCP-1-induced protein (MCPIP1) via the JAK2-STAT3 signaling pathway. Although CCL2 does not directly affect MM cells’ proliferation and chemoresistance, MCPIP1 plays a role in M2 polarization and enhances the protective effect of macrophages in MM. The research indicates that CCL2 expression is linked to therapeutic status and can be a good prognostic factor.

Aside from CCL2, CCL3, known as macrophage inflammatory protein-1α (MIP-1α), can bind to CCR1 to protect MM cells from apoptosis induced by melphalan and bortezomib. Increased CCR1 is associated with upregulated Bcl-2, Bcl- xl, survivin, and downregulated Bim, leading to chemoresistance [21]. MM cells can also induce the differentiation of peripheral blood mononuclear cells (PBMCs) towards MDSCs by secreting CCL5 [22]. In vitro experiments show that the secretion of CCL5 and CCL3 is significantly higher in MDSCs-inducible groups than in MDSCs-non-inducible groups. Moreover, MDSCs-non-inducible groups gained the ability to induce MDSCs by adding CCL5, while supplementation of CCL3 did not promote MDSCs differentiation. The serum level of CCL5 in patients with better response significantly decreased after receiving combination therapy of carfilzomib, lenalidomide (LEN), and dexamethasone, suggesting that CCL5 may promote disease progression. It may be practical to monitor the CCL5 level to manage the disease.

In addition to CCL2 and CCL5, CCL3 shows multifunctional properties that can aggravate the disease. The CCL3-CCR1 axis can attract M2 macrophages into TME, resulting in anemia and metastasis in MM. Research shows that elevated CCL3 levels in the myeloma microenvironment impair erythrocyte differentiation of hematopoietic stem and progenitor cells (HSPCs), leading to anemia [14]. CCL3 suppresses the expression of GATA1 via p38 signaling, which hinders erythroid differentiation in CD34 + HSPCs. The suppressive effect of CCL3 on erythropoiesis can be blocked by CCR1 antagonists, suggesting that CCL3/CCR1/phos-p38 is critical to CCL3-induced anemia in MM. A high level of CCR1 is observed in MM PCs and indicates a poor prognosis [23]. Moreover, CCR1 is an independent prognosis factor of MM patients and induces splenic and bone dissemination of MM cells [18]. The CCL3-CCR1 axis plays a vital role in overcoming retention caused by CXCL12 and the migration of MM cells from the bone environment to peripheral blood [18]. Based on the current comprehension of CCL3, its prognostic and therapeutic value may be worth further exploration.

Some chemokines utilized by MM cells can promote migration and chemoresistance, while others may participate in antigen uptake, cytotoxicity, and proliferation of lymphocytes. For instance, the CCL19-CCR7 axis induces migration of DCs towards sites with a higher concentration of CCL19 and mediates DC homing [24, 25]. DC vaccines utilizing DCs from patients to activate immune responses have been used in clinical practice, and immunogenicity determines DC vaccines’ efficacy. Inspired by the function of CCL19-CCR7, research finds that monocyte-derived DCs have a lower transcript and protein level of CCR7, leading to migration dysfunction [26]. By contrast, hematopoietic stem cell-derived DCs are preferable sources of DC vaccine, as stem cell-derived DCs from MM patients show similarities in cell yield, morphology, and phenotype compared to healthy donors. It indicates that chemokines can also be a possible reference standard to select cells used in autologous cell transplantation.

#### CXC chemokine/receptor subfamily

The CC chemokine subfamily and the CXC chemokine subfamily comprise most of the chemokine family. Like CC chemokines, CXC chemokines are a double-edged sword in MM. While some induce bone destruction [13, 27, 28], chemoresistance [29], and tumor metastasis [30], others can enhance the cytotoxicity of CD8 + T cells or induce apoptosis of MM cells to protect the body against tumors [17].

Chemokines such as CXCL7, CXCL8, CXCL12, and CXCL13 by MM cells and surrounding cells contribute to disease progress and decrease the overall survival in patients [13, 28, 31]. CXCL7, for example, is processed by neutrophils and interacts with CXCR2 to recruit neutrophils [32]. It also plays a role in osteoclastogenesis and the formation of osteoclasts that break down bone tissue, a common characteristic of MM [33].

Matrix metalloproteinase-13 (MMP13) secreted by mesenchymal stromal cells (MSCs) can enhance the activation and formation of osteoclasts by promoting the bioavailability of CXCL7 [13]. However, an in vivo experiment demonstrated that mice with MM cells had elevated MMP-13 in mRNA level, resulting in bone lesions and lower overall survival. Though MMP-13 expression is not observed in all MM patients, analysis shows that it is associated with overall survival. Understanding the role of MMP-13 in regulating CXCL7 bioavailability may provide insight into bone lesions in MM.

CXCL8, also known as interleukin-8 (IL-8), can activate CXCR1 and CXCR2 to recruit neutrophils [27], similar to CXCL7. In patients with MM, CXCL8 levels are higher than in healthy individuals, indicating an association between CXCL8 and MM [34]. Interestingly, there is a difference in IL-8 concentration between males and females worth exploring. Additionally, it has been reported that IL-8 is linked to osteolysis in breast cancer, underscoring its role in complications in MM [35]. In MM, MM cells can secrete exosomes that MSCs can internalize, leading to upregulation of the expression of IL-8 [31]. This process relies on amphiregulin (AREG) packed into exosomes and its interaction with epidermal growth factor receptor (EGFR). According to the study, targeting EGFR may offer a practical and innovative strategy to inhibit the CXCL8-CXCR1/2 axis in MM.

Though studies have proved the role of CXCL13 in several hematologic diseases [36–38], it has been suggested recently that the CXCL13-CXCR5 axis contributes to tumor progress. Higher levels of CXCL13 are not only due to the direct secretion of malignant cells but also bone marrow stromal cells (BMSCs) and macrophages stimulated by malignant cells [28]. MM cells can rely on Bruton’s tyrosine kinase (BTK) signaling to induce M2 polarization, while M2 polarized macrophages mutually upregulate CXCL13 expression in MM cells via TGFβ. With a higher level of CXCL13, enhanced formation of osteoclasts and elevated receptor activator of nuclear kappa B ligand (RANKL) is observed, indicating the role of CXCL13 in osteolytic disease. In addition to lytic lesions, biopsies suggest the relationship between CXCL13 and extramedullary disease. And MSCs have been found to mediate chemoresistance to bortezomib depending on CXCL13 [39]. However, there are currently no small molecule inhibitors directly targeting CXCL13. Future research may attempt to complement the lack of current study due to its function on bone destruction, chemoresistance, and extramedullary disease.

Besides pro-tumor CXC chemokines mentioned above, CXCL12, also known as stromal cell-derived factor-1 (SDF-1), attracts great interest from researchers due to its complex function in MM. CXCL12 can bind to CXCR4 to participate in various dysfunctions in hematological diseases [40, 41]. Emerging studies suggest the role of CXCL12 in multifaceted function in MM, including tumor migration [30] and chemoresistance [29, 42]. CXCL12 has two isoforms, CXCL12alpha and CXCL12gamma, each with distinct stability and immobilization properties [43]. CXCL12alpha is known to promote the phosphorylation of phosphoinositide 3-kinase (PI3K) and protein kinase B (PKB), which further leads to the overexpression of interleukin-6 (IL-6) [42]. Furthermore, the CXCL12alpha-CXCR4 axis contributes to a higher adhesion rate between MM cells and BMSCs, contributing to lower apoptosis in the coculture state. CXCL12gamma, expressed in BMSCs, mediates chemoresistance and requires heparan sulfate proteoglycans (HSPGs) to be immobilized to the membrane [29]. Notably, protection provided by BMSCs functions via adhesion instead of solvable molecules and can be abolished by CXCL12gamma-CXCR4 inhibition. Regardless of chemoresistance, the CXCL12-CXCR4 axis may also induce extramedullary migration [30]. It was known that MM cells could emigrate from bone marrow to form circulating plasma cells (cPCs) and invade other tissues in the past [44]. However, the mechanism is still unclear and remains to be discussed. Recent single-cell sequencing results have revealed increased secretion of CXCL12 in cPCs, indicating its potential role in extramedullary plasmacytoma.

While some CXC chemokines, as mentioned earlier, act as tumor promoters in MM, others, like CXCL4 and CXCL10, impede malignant cell growth. CXCL4, called platelet factor 4 (PF-4), can induce MM cell apoptosis by enhancing SOCS3 to regress STAT3, validating its potential predictive value [45]. A recent study shows that higher PF-4 levels correlate with better outcomes, and higher PF-4 is also observed in healthy individuals [46]. Differently, CXCL10 can recruit natural killer (NK) cells, cytotoxic T lymphocytes, and macrophages, playing a crucial role in anti-tumor immunity through CXCL9/10/11-CXCR3 axes [47]. As CXCL10 is induced by interferon, including IFN-α/β and IFN-γ, it is also named IFN-γ-induced protein-10, and its association with chemotaxis, cytotoxicity, and proliferation is well-documented [48]. CXCL10 has been shown to enhance CAR-T cells’ proliferation and anti-tumor ability in vitro [17]. And patients undergoing CAR-T therapy with higher CXCL10 at baseline reveal better outcomes than those with lower CXCL10. However, MM cells may utilize the CXCRL9/10-CXCR3 axes to interfere with the bone marrow localization of NK cells to evade immune surveillance [49]. Blocking CXCR3 has been shown to improve NK cells’ infiltration in the bone marrow and reinforce IL-15-activated NK cells’ anti-tumor activity [50]. Therefore, due to their complicated functions, the decision to boost or inhibit CXCL9/10-CXCR3 axes should be based on specific clinical backgrounds.

### Therapy based on chemokines

There are two mainstream strategies for utilizing chemokines to treat tumors: targeting pro-tumor chemokines and increasing the concentration of anti-tumor chemokines [51]. Additionally, some studies are exploring anti-tumor chemokines with adoptive cellular therapy (ACT) to enhance its effectiveness. Here, aimed at pro-tumor and anti-tumor chemokines, we will discuss the use of chemokines in treating MM, respectively (Table 1).

**Table 1:** Chemokine network in multiple myeloma

	**Chemokines**	**Receptor**	**Function in MM**	**Value**	**Modulators**	**Reference**
**Pro-tumor**	CCL2	CCR2 CCR4	When MM cells interact with macrophages, they trigger the expression of CCL2, which activates the JAK2/STAT3 pathway and increases MCPIP1 expression in macrophages. This helps to inhibit caspase activation in MM cells and encourages M2 polarization.	The CCL2-CCR2 axis prevents MM cells from apoptosis induced by chemotherapy drugs. CCL2 expression is likely to be tightly linked to treatment status.	Carlumab (tested in idiopathic pulmonary disease, pancreatic cancer, and solid tumors)	[15]
	CCL3 (MIP-1α)	CCR1 CCR5	Elevated levels of CCL3 inhibit the expression of GATA1 and interfere with erythroid differentiation in CD34 + HSPCs via p38 signaling. Additionally, CCL3 elevates the expression of Bcl-2, Bcl-xl, and survivin while decreasing Bim expression, contributing to apoptosis resistance. The CCL3-CCR1 axis is linked to the migration of MM PCs from the bone marrow.	Blocking the CCL3-CCR1 pathway has the potential to alleviate anemia, bone disease, and the dissemination of MM tumors. It can also increase the effectiveness of melphalan and bortezomib in fighting MM and managing its progression.	AZD-4818 (tested in chronic obstructive pulmonary disease) BI-638683 (tested safety) BL5923 (tested in colon cancer and systemic lupus erythematosus) BMS-817399 (tested in RA) BX471 C-6448 (tested in MS) C-4462 (tested in RA) CCX9588, CCX721 CCX354 (tested in RA) CP-481715 (tested in allergic contact dermatitis) MLN-3701/MLN-3897 PS-031291/PS-375179 (discontinued) UCB-35625 (tested in allergic inflammation and HIV-1) J113863 (tested in arthritis)	[14, 18, 21, 50]
	CXCL7	CXCR2	The processing of CXCL7 by MMP13 results in an increase in bioavailable CXCL7 and higher rates of osteoclast formation.	MMP13 activity is associated with reduced overall survival in tumor-bearing mice.	MMP13 inhibitors	[13]
	CXCL8 (IL-8)	CXCR1 CXCR2	When MM-derived exosomes stimulated MSCs, they increased the secretion of IL-8, encouraging osteoclast function through the EGFR pathway. MM cells utilized MIF to induce the expression of IL-8 and IL-6 in BMSCs, but JQ1 was found to be effective in inhibiting cMYC, which helped to suppress this process.	Modulation of IL-8 is a possibility for therapeutic intervention in clinical practice.	CXCR1/2 inhibitor CXCL8 mAb AREG mAb EGFR inhibitors (Gefitinib) JQ1	[31, 34, 52]
	CXCL12 (SDF-1)	CXCR4 CXCR7	Abnormal overexpression of CXCL12 is observed in cPCs, which results in its translocation into the bloodstream. The PI3K/PKB signaling pathway is responsible for upregulating IL-6 expression by CXCL12alpha, leading to reduced apoptosis of MM cells in the cell adhesion state. In addition, CXCL12gamma, immobilized to the membrane of BMSCs by HSPGs, facilitates MM cells’ adhesion to the stromal niche and promotes resistance against proteasome inhibitors.	CXCL12 contributes to the chemoresistance and adhesion of MM cells, while a low level of CXCR4 expression is associated with bortezomib resistance in MM.	Plerixafor Olaptesed Pegol Ruxolitinib Copanlisib Ulocuplumab Motixafortide F50067	[29, 30, 40–42, 53, 54]
	CXCL13	CXCR5	CXCL13 is linked to the expression of RANKL in BMSCs and macrophages and the formation of osteoclasts. MM cells secrete CXCL13 to trigger M2 macrophage polarization and activate osteoclasts.	CXCL13 is associated with bone destruction, growth of tumors, and chemoresistance in MM	Ibrutinib	[28, 39]
**Anti-tumor**	CCL19	CCR7	The CAR-T cells that express IL-7 and CCL19 demonstrate excellent expansion, differentiation, migration, and durability. Moreover, the CCL19-CCR7 axis plays a crucial role in DC migration, and in MM, impaired CCR7 expression in Mo-DCs decreases the migration of DCs.	CAR-T cells expressing IL-7 and CCL19 manifest safety and efficacy and are worthy of further clinical study. SC-DCs with superior migration than Mo-DCs are potential candidates for cancer vaccines.	CAR-T cell immunotherapy DC vaccine	[16, 26]
	CXCL4 (PF-4)	CXCR3	Former research suggests PF-4 can induce MM cell apoptosis by upregulating SOCS3 expression to regress STAT3.	A lower serum level of PF-4 indicates poor overall survival.	-	[46]
**Dual-effect**	CCL5	CCR1 CCR3 CCR4 CCR5	CCL5 secreted by MDSC-inducible HMCLs is essential for MDSC induction in the myeloma microenvironment.	The CCL5-CCR5 axis can be intervened by immunomodulatory drugs acting on MM cells and PBMCs to prevent MDSC induction.	LEN Pomalidomide	[22]
	CXCL9 CXCL10	CXCR3	CXCR3 restrains IL-15-activated NK cells and anti-tumor efficacy from accumulating in the bone marrow.	Targeting CXCR3 can improve NK cell-dependent immunotherapy.	CXCR3 mAb Eldelumab (Targeting CXCL10)	[55]
			CXCL10 recruits CD8 + T cells and promotes proliferation rate, sensitivity, and cytolytic activity in targeting tumor cells.	Adding CXCL10 to CAR-T cells enhances anti-tumor efficacy, while its safety needs further clinical practice.	CAR-T cell immunotherapy	[17]

*Abbreviations*: *MCPIP1* MCP-1-induced protein, *MIP-1α* Macrophage inflammatory protein-1alpha, *BMSCs* Bone marrow stromal cells, *HSPCs* Haematopoietic stem and progenitor cells, *PCs* Plasma cells, *RA* Rheumatoid arthritis, *MS* Multiple sclerosis, *MMP13* Matrix Metalloproteinase-13, *MSCs* Mesenchymal stromal cells, *MIF* Macrophage migratory inhibitory factor, *mAb* Monoclonal antibody, *EGFR* Epidermal growth factor receptor, *AREG* Amphiregulin, *cPCs* Circulating plasma cells, *SDF-1* Stromal cell-derived factor-1, *PI3K* Phosphoinositide 3-kinase, *PKB* Protein kinase B, *HSPGs* Heparan sulfate proteoglycans, *RANKL* Receptor activator of nuclear kappa B ligand, *CAR* Chimeric antigen receptor, *Mo-DCs* Monocyte-derived dendritic cells, *SC-DCs* Stem cell-derived dendritic cells, *NK* Natural Killer, *DC* Dendritic cell, *PF-4* Platelet factor 4, *MDSC* Myeloid-derived suppressor cell, *HMCLs* Human myeloma-derived cell lines, *PBMCs* Peripheral blood mononuclear cells

#### Targeting pro-tumor chemokines

Malignant cells can trigger other cells or autonomously secrete chemokines to evade immune supervision. This creates an environment known as TME that protects MM cells from immune cells, making them less susceptible to treatment and leading to refractoriness. Thus, clinical practices focus on blocking signaling between MM and normal cells with inhibitors or small molecules to break down these barriers.

Chemokines such as CCL2, CCL3, CCL5, and CXCL12 contribute to immune suppression in MM, leading to chemoresistance. The CCL2-CCR2 axis is responsible for M2 polarization and prevents MM cells from apoptosis caused by chemotherapy drugs [15]. Studies are currently being conducted to address this issue by targeting the CCL2-CCR2 axis associated with the recruitment of TAMs. For instance, carlumab has shown anti-tumor activity in preclinical and clinical trials. However, subsequent tests have shown no long-term suppression of serum CCL2 or significant anti-tumor effects [56].

Considering the protective effect of CCL3 on malignant cells, researchers have combined CCL3-neutralizing antibodies with melphalan and bortezomib to enhance cytotoxicity in MM cells [21]. Inspired by the complicated impact of the CCL3-CCR1 axis, various CCR1 antagonists have been developed to block the CCL3-CCR1 axis, although their effectiveness requires further evaluation [50]. It is revealed that BX471, an antagonist of CCR1, can reverse the reduced erythropoiesis induced by the CCL3-CCR1 axis in ex vivo [14]. Moreover, another CCR1 antagonist, CCX9588, has been proven to prevent malignant cells from migrating to CCL3 in vitro and disseminating to the bone in vivo [18]. Though most modulators are preclinical, CCL3 and CCR1 are potential therapeutic targets in MM and are worth exploring.

Though CCL2 and CCL3 may be potential therapeutic targets in MM, antibodies or small molecules aimed at them have yet to be widely applied in clinical practice. Notably, inhibition of CXCL12 has been confirmed as an efficient approach to managing MM patients and is currently being used. Among CXCR4 antagonists, plerixafor (AMD3100) is the first and the only chemokine modulator approved for treating multiple myeloma patients. Although initially used in stem cell mobilization [8], plerixafor has undergone phase I/II clinical trial with safety and efficacy in combination with bortezomib [6]. Despite investigating the new use of plerixafor, new modulators are being developed and in clinical trials. CXCR4 antagonist motixafortide (BKT140) reveals satisfying outcomes in mobilizing stem cells in phase III trial and is likely to be popularized in the treatment [9]. Besides, the phase Ib/II trial of ulocuplumab also received exhilarating results [7]. Inspired by the success of plerixafor and ulocuplumab and the complicated role of the CXCL12-CXCR4 axis in MM, more efforts are made to explore inhibitors, antagonists, and antibodies aimed at the interaction between CXCL12 and its receptor. Olaptesed pegol (NOX-A12), which can bind to CXCL12, shows benefits in combination with dexamethasone, indicating the strategy to target CXCL12 rather than CXCR4 [57].

Unlike CCL2, CCL3, and CXCL12, CCL5 regulates the formation of MDSCs. To prevent the protective effects of CCL5 on MM cells, immunomodulatory drugs are used to block the CCL5-CCR5 axis and interfere with MDSCs induction [22]. LEN and pomalidomide can downgrade the expression of CCR5 and increase interferon regulatory factor 8 (IRF8) in the mRNA level in peripheral blood mononuclear cells (PBMCs) while also hindering the expression of CCL5 in MM cells. By acting on both normal PBMCs and malignant cells, these drugs can decrease the protection of MDSCs against MM cells and improve disease progression.

In addition to abating chemoresistance in MM, targeting pro-tumor chemokines can relieve bone destruction. CXCL8 participants in osteolytic lesions in MM, so antibodies targeting CXCL8 have been developed to abrogate the CXCL8-CXCR1/2 axis [58–60]. However, CXCL8 antibodies have yet to be applied in managing MM patients. Despite small molecules or antibodies directly binding to CXCL8 or CXCR1/2, targeting related signaling to reduce CXCL8 secretion is also feasible. Gefitinib, an EGFR inhibitor, can block AREG-EGFR signaling to relieve bone destruction in MM [31, 61], while JQ1 acts on BMSCs to disturb CXCL8 synthesis and reveal anti-tumor efficacy [52].

Instead of directly focusing on the concentration of chemokines, another perspective considers regulating their bioavailability. As MMP13 regulates the bioavailability of CXCL7, Lo et al. conducted both cell and animal experiments to explore the efficacy of MMP13 inhibitors [13]. The result indicated that MMP13 inhibitors reduced osteoclastogenesis and restrained the growth of malignant cells, leading to improved overall survival. Unlike generally inhibiting CXCL7 or CXCR2, the mechanism of CXCL7 bio-utilization provides a new view to block the CXCL7-CXCR2 axis. Besides using inhibitors or small molecules to interfere with the axis, it is practical to decrease the bioavailability of CXCL7 to improve bone lesions in MM. Some studies on the CXCL12-CXCR4 axis also attempt to regulate CXCL12 concentration via associated signaling pathways. For example, ruxolitinib can block the JAK1/2 pathway and further downgrade the expression of CXCL12 in monocytes and CXCR4 in MM cells when coculturing [53]. Similarly, some studies utilize inhibitors to block responses induced by the CXCL12-CXCR4 axis. Copanlisib, a PI3K inhibitor, can interfere with CXCL12-dependent chemotaxis to reduce fibroblast migration and restrict MM cell chemoresistance [54]. According to the complicated biological function of the CXCL12-CXCR4 axis in MM, further exploration may unveil more signaling pathways that interact with the axis and provide additional potential targets to abate pro-tumor effects.

Studies on CXCL13 have mainly focused on indirect regulation rather than directly targeting CXCL13 or its receptor, CXCR5, alongside CXCL7 and CXCL12. As the interaction between MM cells and macrophages depends on BTK, it is also a promising approach to inhibit pro-inflammatory reactions in macrophages. Ibrutinib, a BTK inhibitor, can reduce abnormal overexpression of CXCL13 [28] in vivo experiments, and the clinical practice achieved satisfying safety and efficacy [62]. Future studies may utilize BTK inhibitors, including ibrutinib, to improve the outcome of patients with refractory MM.

#### Increasing the concentration of anti-tumor chemokines

Despite decreasing the concentration of pro-tumor chemokines, increasing the concentration of anti-tumor chemokines is also a practical strategy in MM treatments. As some chemokines contribute to eliminating tumors, clinical researchers have focused on arming CAR-T cells with anti-tumor chemokines to enhance their potency in removing malignant cells.

One way to utilize anti-tumor chemokines is by increasing the concentration of CCL19, which is associated with antigen-presenting. In addition to using stem-derived DCs instead of monocyte-derived DCs [26], arming CAR-T cells with CCL19 to enhance cytotoxicity is also worth exploring. CAR-T cells secreting CCL19 and IL-7 have been proven to have higher infiltration of DCs and T cells in tumor tissue [63]. While in MM, Duan et al. designed B-cell maturation antigen (BCMA)-7 × 19 CAR-T cells, which overexpressed CCL19 and IL-7 to cure two patients with refractory MM [16]. BCMA-7 × 19 CAR-T cells revealed delayed terminal differentiation, leading to a higher ratio of stem cell-like memory T cells (Tscms) and durability. Although with more potent cytotoxicity towards tumor cells, BCMA-7 × 19 CAR-T cell therapy was safe with self-limiting and revisable adverse effects. Though the clinical trial had a small sample of only two patients, it is valuable to dig out the potential of BCMA-7 × 19 CAR-T cells in MM management.

Similarly, the addition of CXCL10 has been shown to enhance CAR-T cells’ proliferation, cytotoxicity, and chemotaxis, making it a possible solution for the remaining challenges of CAR-T therapy. CXCL10 can reduce PD-1 expression in CAR-T cells and provides a viable solution to improve the exhaustion of CAR-T cells [17]. However, CXCL10 may adversely affect NK cell localization and potential cytotoxicity, attenuating the efficacy of therapies based on NK cells. Limited by its negative effect on NK cell localization and potential cytotoxicity, the application of CXCL10 to CAR-T therapy may have a long way to go. In a word, exploring anti-tumor chemokines can provide CAR-T therapy with novel targets and strategies to solve current challenges in the infiltration and exhaustion of CAR-T cells.

Interactions between chemokines and their receptors interweave a complex network in MM. Blue boxes in the figure indicate the anti-tumor effects of chemokines, while red boxes indicate the pro-tumor effect of chemokines. Malignant cells can utilize the chemokine network to interfere with physiological functions, including erythroid differentiation, osteoclast, and M2 polarization, induce chemoresistance, and downregulate the immune system. MΦs, PBMCs, MDSCs, BMSCs, MSCs, and HSPCs can be influenced and participate in the pathological progression. MM cells can use exosomes, MMPs, HSPGs, or MIF to regulate chemokines and foster TME suitable for themselves. Conversely, chemokines also aid immune cells in migrating into tumor sites, enhancing the immune system and inducing apoptosis of malignant cells. And such function may be applied to develop drugs against MM. MM, multiple myeloma; MΦs, macrophages; PBMCs, peripheral blood mononuclear cells; MDSCs, myeloid-derived suppressor cells; BMSCs, bone marrow stromal cells; MSCs, mesenchymal stromal cells; HSPCs, hematopoietic stem and progenitor cells; MMPs, matrix metalloproteinases; HSPGs, heparan sulfate proteoglycans; MIF, macrophage migratory inhibitory factor; TME, tumor microenvironment.

#### Future and challenges of chemokines

Interactions between chemokines and their receptors are complex and correlate with pathological progression and prognosis of diseases. Except for MM, chemokine modulators are developed to heal various conditions such as pulmonary fibrosis [64], colon cancer [65], multiple sclerosis (MS) [66], and rheumatoid arthritis (RA) [67–69], indicating the potential of chemokines as drug targets.

**Fig. 3:**
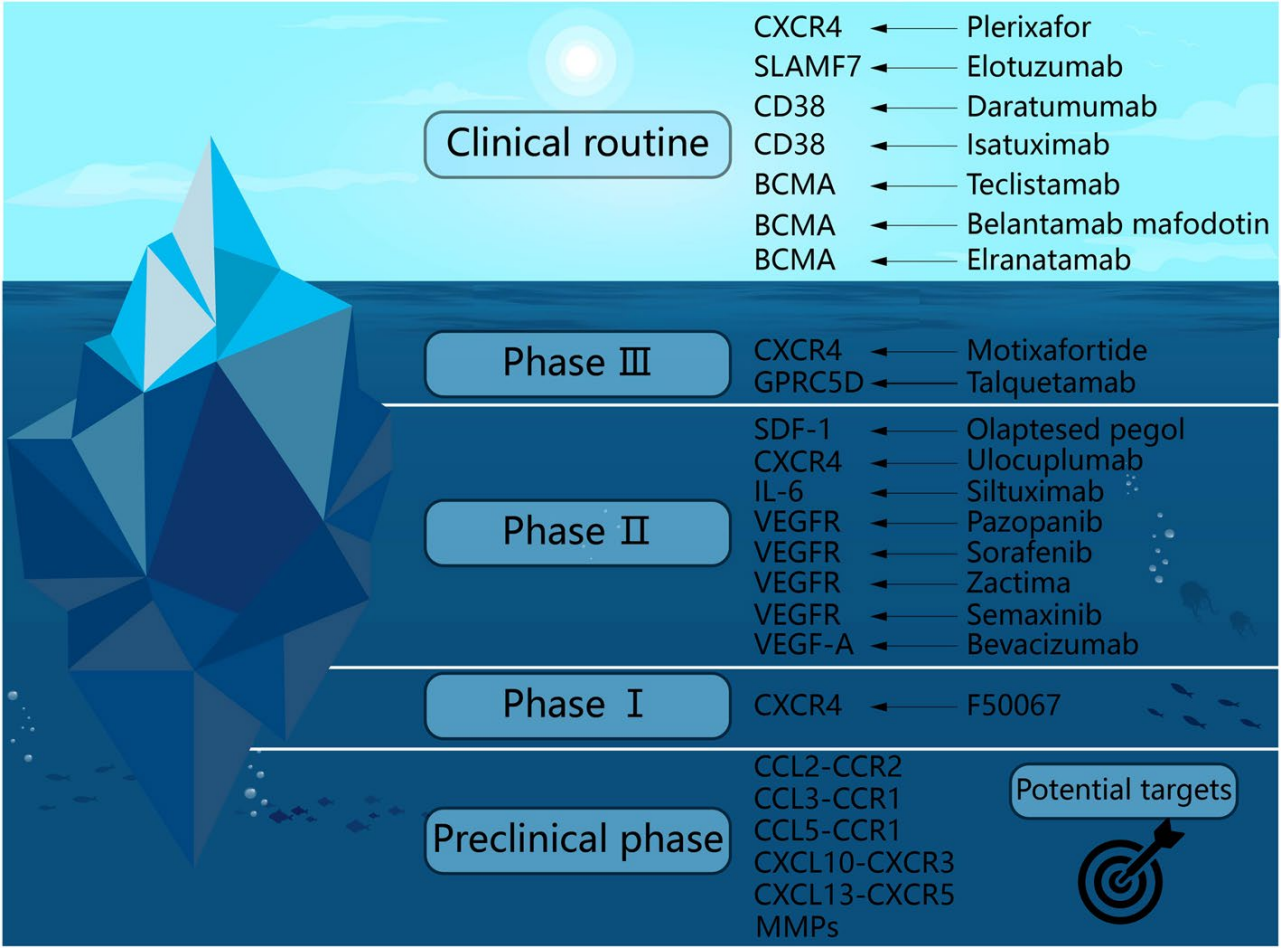
Target inhibitors, antagonists, or antibodies in multiple myeloma

Iceberg theory is quite suitable for describing modulators’ developments in MM (Fig. 3). IL-6, matrix metalloproteinases (MMPs), and vascular endothelial growth factor (VEGF) are potential targets in MM, which lay in part close to the water surface. Though inhibition in clinical trials did not receive satisfying outcomes [70–76], further studies may attempt to find specific patients sensitive to these modulators, explore combinations with other drugs, or develop more efficient modulators. In contrast, G protein-coupled receptor class C group 5 member D (GPRC5D) [77], CD38 [78, 79], signaling lymphocytic activation molecule F7 (SLAMF7) [80–82], and BCMA [83–85] showed favorable results. Modulators targeting them are in the upper layers; some are already in clinical use (Table 2).

**Table 2:** Some target inhibitors, antagonists, or antibodies in multiple myeloma

**Targets**	**Modulators**	**Function**	**Investigational stage**	**Results**	**Reference**
CXCL12-CXCR4	Olaptesed pegol (NOX-A12)	CXCL12 (SDF-1) inhibitor Inhibiting dissemination and colonization of MM cells	Phase II	Confirmed safety and efficacy at least 72 h	[57, 86]
	F50067	CXCR4 antagonist Sensitizing MM cells to LEN and dexamethasone Inducing CDC and ADCC	Phase I	Observed dose-limiting toxicity	[87]
	Plerixafor (AMD3100)	CXCR4 antagonist Sensitizing MM cells to bortezomib Mobilizing hematopoietic cells	Phase II	Confirmed safety and efficacy in combination with bortezomib in MM	[6]
			Phase IV	Confirmed safety and efficacy in improving mobilization of aHSCs.	[8]
	Ulocuplumab	CXCR4 monoclonal antibody Improving overall response rate to standard therapy	Phase II	Confirmed safety and efficacy in combination with either LEN and dexamethasone or bortezomib and dexamethasone in MM	[7]
	Motixafortide (BKT140)	CXCR4 antagonist Mobilizing hematopoietic cells	Phase III	Confirmed safety and efficacy of mobilization in MM	[9]
IL-6	Siltuximab (CNTO 328)	IL-6 monoclonal antibody Inhibiting smoldering MM from transiting to MM	Phase II	Confirmed safety and failure to meet the desired effect	[70]
VEGF	Zactima (ZD6474)	VEGFR and EGFR inhibitor Inhibiting angiogenesis	Phase II	Confirmed safety while no reduction of M protein	[71]
	Sorafenib (BAY 43-9006)	Raf-kinase and VEGFR inhibitor Inhibiting Raf-signaling pathway and angiogenesis	Phase II	No activity by the International Uniform Response Criteria for MM	[72]
	Bevacizumab	VEGF-A monoclonal antibody Inhibiting angiogenesis	Phase II	No significant difference between combination therapy and single-agent thalidomide or single-agent bortezomib	[73, 74]
	Semaxinib (SU5416)	RTKI of VEGFR-2 Inhibiting VEGF-induced angiogenesis	Phase II	Minimal clinical activity	[75]
	Pazopanib	VEGFR, PDGFR, and c-Kit inhibitor Inhibiting angiogenesis	Phase II	No meaningful clinical responses in the single-agent treatment	[76]
CD38 (Only post-phase III modulators are listed.)	Daratumumab	CD38 monoclonal antibody Sensitizing MM cells to proteasome inhibitors	Phase IV	Confirmed benefits in combination with carfilzomib and dexamethasone	[78]
	Isatuximab	CD38 monoclonal antibody Inducing MM cells’ apoptosis	Phase IV	Confirmed benefits in combination with carfilzomib and dexamethasone	[79]
SLAMF7	Elotuzumab	SLAMF7 monoclonal antibody Inducing ADCC, ADPC and inhibiting adhesion of MM cells	Phase IV	Confirmed benefits in combination with LEN and dexamethasone	[80–82]
BCMA (Only post-phase III modulators are listed.)	Teclistamab	BCMA and CD3 bispecific antibody Inducing T cells’ activation and MM cells’ apoptosis	Phase IV	Approved treatments in relapsed or refractory MM	[83]
	Belantamab mafodotin	ADC targeting BCMA Inducing MM cells’ apoptosis and activation of anti-tumor immune responses	Phase IV	Approved treatments in pluri-refractory patients	[85]
	Elranatamab	BCMA and CD3 bispecific antibody Inducing T-cell mediated cytotoxicity	Phase IV	Observed promising early responses with manageable safety	[84]
GPRC5D	Talquetamab	CD3 and GPRC5D bispecific antibody Mediating immune cells to attack GPRC5D-expressing MM cells	Phase III	Confirmed response and safety in treating relapsed or refractory MM in phase II trial	[77]

*Abbreviations*: *SDF-1* Stromal Cell-derived Factor-1, *MM* Multiple myeloma, *LEN* Lenalidomide, *aHSCs* Autologous hematopoietic stem cells, *CDC* Complement-dependent cytotoxicity, *ADCC* Antibody-dependent cellular cytotoxicity, *VEGFR* Vascular endothelial growth factor receptor, *EGFR* Endothelial growth factor receptor, *VEGF* Vascular endothelial growth factor, *RTKI* Tyrosine kinase inhibitor, *PDGFR* Platelet-derived growth factor receptor, *SLAMF7* Signaling lymphocytic activation molecule F7, *ADPC* Antibody-dependent cellular cytotoxicity, *BCMA* B-cell maturation antigen, *ADC* Antibody-drug conjugate, *GPRC5D* G protein-coupled receptor class C group 5 member D

Among drugs aimed at chemokines, only drugs targeting the CXCL12-CXCR4 axis have been used in clinical practice. In contrast, most neutralizing antibodies, antagonists, or inhibitors are still in the stage of experiment or development. Plerixafor is at the tip of the iceberg, which has been uncovered and applied already, while motixafortide has passed the phase III trial and will surface. Early studies mainly utilized plerixafor to mobilize CD34 + hematopoietic cells for ACT [8]. Aimed at reducing the failure of mobilization of autologous hematopoietic cells, motixafortide, known as BKT140, has been developed and has passed phase III study recently [9], which may be a substitution for plerixafor. As scientists have a deeper insight into the CXCL12-CXCR4 axis in MM, a phase I/II clinical research innovatively combined plerixafor with bortezomib to sensitize MM cells and improve outcomes [6]. In addition to plerixafor, ulocuplumab, another CXCR4 antagonist, is also proven efficient in increasing the response rate in refractory MM in phase II clinical study [7]. Regarding the CXCL12-CXCR4 axis, some modulators targeting CXCL12 instead of CXCR4 are also worth further exploring. Olaptesed pegol is a CXCL12 inhibitor that could improve response rates in combination with bortezomib and dexamethasone [57].

Except for CXCL12-CXCR4, CCL3-CCR1 is another hotspot axis, whereas modulators targeting CCR1 still have a long way to go. Given that CCL3-CCR1 participates in inflammation, various antagonists or inhibitors of CCR1 are developed to cure diseases associated with immune disorders. Among them, only CCX354, which is designed for RA, passed phase II [67]; nevertheless, AZD-4818 [88], BMS-817399 [89], and CP-481715 [90] are proved to be either toxic in phase I or limited efficacy in phase II. Besides, most CCR1 antagonists remain uncertain of their efficacy and are in the preclinical phase or phase I [18, 65, 91–97]. Thus, modulating targeting CCL3-CCR1 resembles the enormous part of the iceberg buried undersea and has great potential to explore. Considering the recent discovery of CXCL13-CXCR5 and the lack of specific inhibitors, the CXCL13-CXCR5 axis is also worth unearthing. Other chemokine-chemokine receptor axes, including CCL5-CCR1, CCL2-CCR2, and CXCL9/10-CXCR3, are also under study, though results revealed the need for improvements of their modulators [55, 56, 64, 66]. Inspired by the newly developed therapy of plerixafor and the appearance of motixafortide, future studies may either invent new modulators targeting chemokines to regulate their biological function or propose innovative uses of drugs approved for market.

Exosomes or proteinases also associates with chemokines [13, 31]. Immunomodulators are proven to participate in regulating the chemokine network [22], while clinical practices that apply anti-tumor chemokines to improve CAR-T therapy demonstrate the feasibility of combining chemokines with ACT [16]. With profound knowledge, more available drugs and treatments would emerge and benefit MM patients. More efforts should be made to probe into the function and pathological mechanism of chemokines in MM to better support the development of new applications of chemokines.

## Conclusion

As chemokines regulate the migration of immune cells, targeting chemokines may provide a possible solution to remodel the TME, activate immunoreaction, and promote the clearance of malignant cells. Studies have tried to block the interaction of pro-tumor chemokine ligands and receptors to alleviate the complications and progress in MM. However, chemokines can interact with multiple receptors to take effect. When antagonists or inhibitors block specific receptors, chemokines may bind to alternative receptors to activate related signaling pathways. Thus, some inhibitors or antagonists targeting single chemokine ligands or receptors may not achieve the expected effect. Combining these inhibitors or antagonists with other drugs is a promising strategy to improve their efficacy. By contrast, increasing anti-tumor chemokines to enhance the clearance of tumor cells is another practical strategy. Chemokines can recruit and activate immune cells, which makes it a possible solution for the exhaustion of CAR-T cells. Arming CAR-T cells with anti-tumor chemokines reveals feasibility and is worth further exploration [16]. Considering exosomes or proteinases are also associated with chemokines [13, 31], research on chemokines can inspire the development of other inhibitors in MM, which may contribute to more potential targets. Finally, probing into chemokines will supplement the mechanism of the current treatment, such as immunomodulators, and better guide the clinical practice. To utilize chemokines to treat MM, more research focused on the related signaling pathways of chemokines should be done. With a comprehensive knowledge of chemokine/chemokine receptor axes, researchers can better take advantage of chemokines to relieve and treat MM.

CXCR4 inhibitor plerixafor is the only chemokine modulator put into clinical use, while motixafortide has passed phase III trial recently. Modulators targeting CXCL12(SDF-1)-CXCR4 have promising potential in development and clinical use. Although F50067, a CXCR4 antagonist, was observed to be toxic in the phase I study, other modulators targeting CXCR4 and CXCL12 reveal safety and efficacy in clinical tests. Other modulators targeting BCMA, CD38, and SLAMF7 are also approved for treating MM patients. Talquetamab, a GPRC5D inhibitor, is in the phase III trial now. By comparison, there are some difficulties in developing VEGF inhibitors with limited efficacy. Inhibition of targets such as CCL3-CCR1, CCL5-CCR1, CXCL10-CXCR3, CXCL13-CXCR5, MMPs, and CCL2-CCR2 may also be developed and applied.

## Data Availability

Not applicable.

## Abbreviations

MM: Multiple myeloma
PCs: Plasma cells
C: Cysteine
TME: Tumor microenvironment
MΦs: Macrophages
Tregs: Regulatory T cells
DCs: Dendritic cells
TAMs: Tumor-associated macrophages
MDSCs: Myeloid-derived suppressor cells
MCP-1: Monocyte chemoattractant protein-1
MCPIP1: MCP-1 induced protein
MIP-1α: Macrophage inflammatory protein-1α
PBMCs: Peripheral blood mononuclear cells
LEN: Lenalidomide
HSPCs: Hematopoietic stem and progenitor cells
MMP13: Matrix metalloproteinase-13
MSCs: Mesenchymal stromal cells
IL-8: Interleukin-8
AREG: Amphiregulin
EGFR: Epidermal growth factor receptor
BMSCs: Bone marrow stromal cells
BTK: Bruton’s tyrosine kinase
RANKL: Receptor activator of nuclear kappa B ligand
SDF-1: Stromal cell-derived factor-1
PI3K: Phosphoinositide 3-kinase
PKB: Protein kinase B
IL-6: Interleukin-6
HSPGs: Heparan sulfate proteoglycans
cPCs: Circulating plasma cells
PF-4: Platelet factor 4
NK: Natural killer
ACT: Adoptive cellular therapy
IRF8: Interferon regulatory factor 8
PBMCs: Peripheral blood mononuclear cells
CAR: Chimeric antigen receptor
BCMA: B-cell maturation antigen
Tscms: Stem cell-like memory T cells
MIF: Macrophage migratory inhibitory factor
RA: Rheumatoid arthritis
MS: Multiple sclerosis
mAb: Monoclonal antibody
Mo-DCs: Monocyte-derived dendritic cells
SC-DCs: Stem cell-derived dendritic cells
HMCLs: Human myeloma-derived cell lines
VEGF: Vascular endothelial growth factor
MMPs: Matrix metalloproteinases
GPRC5D: G protein-coupled receptor class C group 5 member D
SLAMF7: Signaling lymphocytic activation molecule F7
CDC: Complement-dependent cytotoxicity
ADCC: Antibody-dependent cellular cytotoxicity
aHSCs: Autologous hematopoietic stem cells
VEGFR: Vascular endothelial growth factor receptor
RTKI: Tyrosine kinase inhibitor
PDGFR: Platelet-derived growth factor receptor
ADPC: Antibody-dependent cellular cytotoxicity
ADC: Antibody-drug conjugate
